# Extinction threatens to cause morphological and ecological homogenization in sharks

**DOI:** 10.1126/sciadv.aea0278

**Published:** 2025-10-29

**Authors:** Mohamad Bazzi, Warwick L. Lloyd, Jun A. Ebersole, Phillip C. Sternes, Jood A. Al Aswad, Jonathan L. Payne

**Affiliations:** ^1^ Department of Earth and Planetary Sciences, Stanford University, 450 Jane Stanford Way, Stanford, CA 94305, USA.; ^2^ McWane Science Center, 200 19th Street North, Birmingham, AL 35203, USA.; ^3^ Education and Conservation Department, SeaWorld, San Diego, CA 92109, USA.; ^4^ Shark Measurements, London, UK.; ^5^ Department of Geosciences, Virginia Tech, Blacksburg, VA 24061, USA.

## Abstract

Global shark biodiversity is in decline, with numerous species facing extinction because of anthropogenic influence. Loss of species richness is expected to diminish trait diversity, encompassing ecological roles and physiological adaptations. We investigate whether the extinction of threatened species, as classified by the International Union for Conservation of Nature, drives morphological and ecological homogenization within *Carcharhinus*, a speciose genus of requiem sharks. We assembled a dataset of tooth morphology from 30 species and combined it with functional data such as diet, habitat, and body size. Simulated extinction scenarios, where species were sequentially removed from the highest to lowest threat level, revealed that the loss of threatened species would result in marked homogenization of morphology and ecology. Along this extinction trajectory, trait structures become increasingly depauperate, marked by contracting depth ranges and declining body-size diversity. Our results indicate that the diverse dental morphologies, shaped over millions of years, are at risk of disappearing—eroding the genus’s capacity to support varied ecological roles.

## Teaser

The selective extinction of threatened shark species is poised to drive substantial morphological and ecological homogenization.

## INTRODUCTION

Biodiversity encompasses more than just species counts. It also includes ecological dimensions such as shape and function (*1*, *2*), as well as evolutionary components like phylogenetic richness (*3*). Consequently, ecosystem responses to extinction cannot be predicted from extension intensity alone [e.g., (*4*–*6*)]. Notably, the fossil record narrates a story of selective survival of species, a phenomenon that has been explained by both chance and/or adaptability of species to withstand changes in their environment (*7*–*10*). The latter explanation emphasizes identifiable traits (ranging from physiological to ecological) (*8*, *11*, *12*) as the basis for why some species become extinct while others survive. The degree of specialization versus generalization has also been hypothesized to influence a species’ ability to survive extinction events (*13*, *14*). For example, highly specialized species, such as certain pollinator-dependent plants or obligate predators, may be more vulnerable, whereas generalist species often exhibit greater resilience [e.g., (*15*, *16*)].

The threats faced by modern species, while not identical to those faced by their ancient counterparts (e.g., bolide impact at the end-Cretaceous mass extinction, 66 million years ago), are nonetheless important for its selectivity as well as its magnitude (*7*, *17*). Among marine vertebrates, sharks and their close relatives (collectively referred to as elasmobranchs) have suffered protracted population declines because of human activity and mainly through chronic overfishing (*18*–*22*). Conservation assessments on this group suggest that nearly one-third of elasmobranch species are already threatened with extinction (*20*). Functionally and ecologically distinct shark species are the most threatened (e.g., large pelagic predators), setting the stage for potential attenuation of trait diversity (*18*, *20*, *23*–*25*). The selective extinction of such species could reduce ecosystem services and create functional gaps in ecosystems (*18*, *23*, *26*). This situation warrants a trait-focused approach to understanding the risk of species extinction as its consequences are likely to affect human economic and cultural structures (*27*–*30*). Furthermore, the loss of unique trait combinations as expressed in morphology could mark the beginning of a decline in morphological variation (i.e., disparity) and in turn result in homogenized phenotypes with constrained functional and ecological utility [e.g., (*31*–*33*)].

Understanding whether extinction filters species toward greater similarity in shape and size, and how this influences ecological diversity, requires measuring anatomical features that ideally also correlate with key lifestyle traits such as feeding, body size, and habitat use (*25*, *31*, *34*–*36*). Such an approach has been conspicuously absent for cartilaginous fishes, in general, and sharks, in particular. Tooth morphology in sharks offers both taxonomic insights (*37*–*39*) and information on a wide range of ecological and functional adaptations (*34*, *40*, *41*). The ease of sampling and quantifying tooth shape lends it to multivariate analysis [e.g., (*34*, *42*–*44*)]. Exploring patterns in the dental morphospace might reveal how extinction threat is distributed with respect to functional roles (*43*, *45*–*47*). Such information is useful to conservation action plans by identifying how extinction threats will alter ecosystem composition and function (*31*, *48*).

Here, we explore the morphological diversity and extinction risk of *Carcharhinus*, a speciose and ecologically prolific genus of requiem sharks (family Carcharhinidae) (*37*, *38*, *49*, *50*). Members of this genus occupy diverse habitats, ranging from coral reefs (e.g., *C. amblyrhynchos*, *C. melanopterus*, and *C. perezi*) (*51*, *52*) to the open ocean (e.g., *C. longimanus*) and even freshwater environments (e.g., *C. leucas*) (*49*). This genus forms a paraphyletic group whose fossil record extends back to the Middle Eocene, around 50 million years ago (*53*), and includes ~35 extant species (*49*). Assessments by the International Union for Conservation of Nature (IUCN) Red List of Threatened Species (www.iucnredlist.org/; last accessed March 2025) show that the vast majority of *Carcharhinus* species is assessed as threatened (17% Critically Endangered, 23% Endangered, and 31% Vulnerable). Their importance in commercial and artisanal fisheries continues to adversely influence their conservation status (*49*, *54*–*56*) and to disproportionately affect medium-to-large–sized and reef-associated species (*18*, *51*, *57*). Their cosmopolitanism, conservation sensitivity, and role as meso- to apex predators make them ideal for investigating the potential ecological consequences of extinction (and even of substantial population reduction). To quantify how the loss of threatened *Carcharhinus* species would affect morphological and ecological diversity, we compiled a comprehensive dataset of tooth shape (*n* = 1256) from 30 *Carcharhinus* species (85.7% of the species diversity) and analyzed threat with respect to shape as well as other trait variables, including diet, habitat, depth, and body size. These sharks are notable for the uniformity of their body form design (*37*, *52*, *58*, *59*), making field identification sometimes difficult (*38*, *60*, *61*). However, the teeth of these sharks (particularly those within the upper dentition) can often be used to distinguish among species (*37*, *38*, *62*). Beyond aiding in species identification, dental characters and tooth shape in sharks correlate with feeding ecology and functional richness (*34*, *63*).

By adopting a combined geometric morphometric and ecomorphological approach, we aim to determine whether threatened and nonthreatened species occupy distinct areas of morphospace, in turn reflecting potential ecological divergence. Under the current threat reality, we anticipate that the extinction of threatened species would erode the reservoir of phenotypic variability (i.e., dispersion and area of space occupied) and simultaneously impoverish other aspects of trait diversity including the range of ecological guilds, leading to functional homogenization across the genus. Our results provide a complementary view to extinction dynamics in modern times and its impact on morphological diversity in extant sharks.

## RESULTS

### Extinction threat in the morphospace

**Fig. 1.**
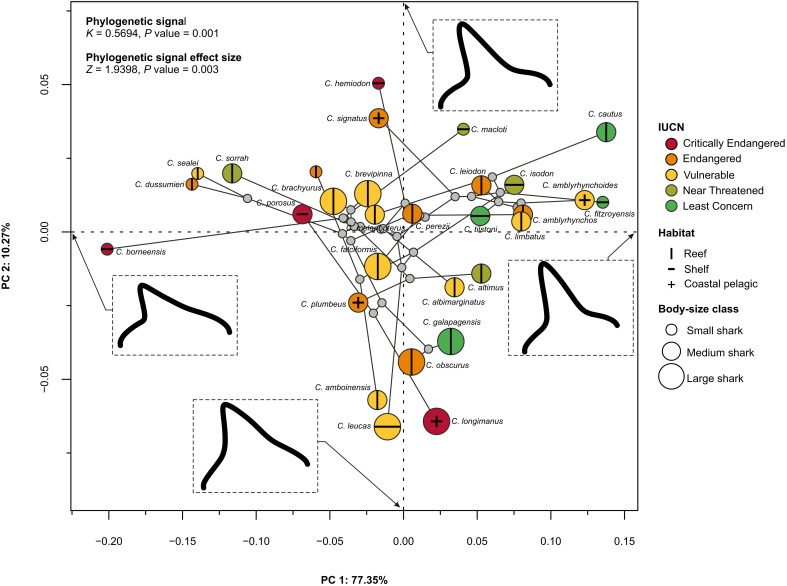
Dental phylomorphospace of the genus *Carcharhinus*. Points are color coded on the basis of IUCN status and scaled relative to the categorical designations of small (<150 cm)–, medium (150 to 300 cm)–, and large (>300 cm)–bodied species [sensu Dulvy *et al.* (*18*)]. Habitats are indicated by symbols (vertical bar, plus and minus signs). Thin-plate spline deformation grids indicate the theoretical shape at the extremes of PC1 and PC2. The phylogenetic signal and effect size estimates for Procrustes shape data are provided.

To gauge the distribution of extinction threat in the shape space, we first ordinated the Procrustes coordinates of species averages using a principal components analysis (Fig. 1). The first axis (PC1: 77.35%) describes a morphological continuum ranging from obliquely cusped and short tooth crowns loading negatively to more elongated, triangular main cusps with wider crown bases, loading positively (Fig. 1). The second axis (PC2: 10.27%) describes broad, triangular tooth crowns with a moderate apical slant negatively to obliquely cusped, well-differentiated main cusps with rounded distal shoulders. Mapping the extinction threat status of species onto the phylomorphospace shows that Critically Endangered species mainly occupy the extremities on both principal component (PC) axes (Fig. 1). These include the shelf-associated, small-bodied *C. borneensis* and *C. hemiodon* and the large, stocky pelagic *C. longimanus*. Endangered and Vulnerable species occupy comparable areas of morphospace along PC1, whereas Near-Threatened species gravitate toward positive values (with the notable exception of *C. sorrah*, which extends into negative areas on PC1). The Least Concern category includes small- to large-bodied species (*C. cautus*, *C. fitzroyensis*, *C. galapagensis*, and *C. tilstoni*) with both reef and shelf habitat associations and occupies positive values on PC1.

Statistical analysis using species averages showed no significant difference in morphospace occupation (i.e., across all axes of variation) among IUCN categories (*R*^2^ = 0.22, *F* = 1.86, *Z* = 1.32, *P* = 0.08) (see the Supplementary Materials for model results obtained from using the entire specimen-level dataset). Yet, pairwise comparisons revealed some differentiation, particularly between Critically Endangered and Least Concern species (*Z* = 2.34, *P* = 0.006) but also between Endangered and Least Concern species (*Z* = 1.70, *P* = 0.04) and Vulnerable and Least Concern species (*Z* = 1.66, *P* = 0.04). A separate linear model, evaluating species as threatened versus nonthreatened, revealed a statistically significant effect (*R*^2^ = 0.12, *F* = 3.87, *Z* = 1.72, *P* = 0.04). Sensitivity analyses revealed that species averages based on upper jaw teeth provide the closest match to the combined shape space ordination (figs. S1 and S2). Last, a moderate yet statistically significant phylogenetic signal in tooth morphology was recovered across our main species-level analyses (Fig. 1 and figs. S1 and S2).

### Erosion of morphospace

**Fig. 2.**
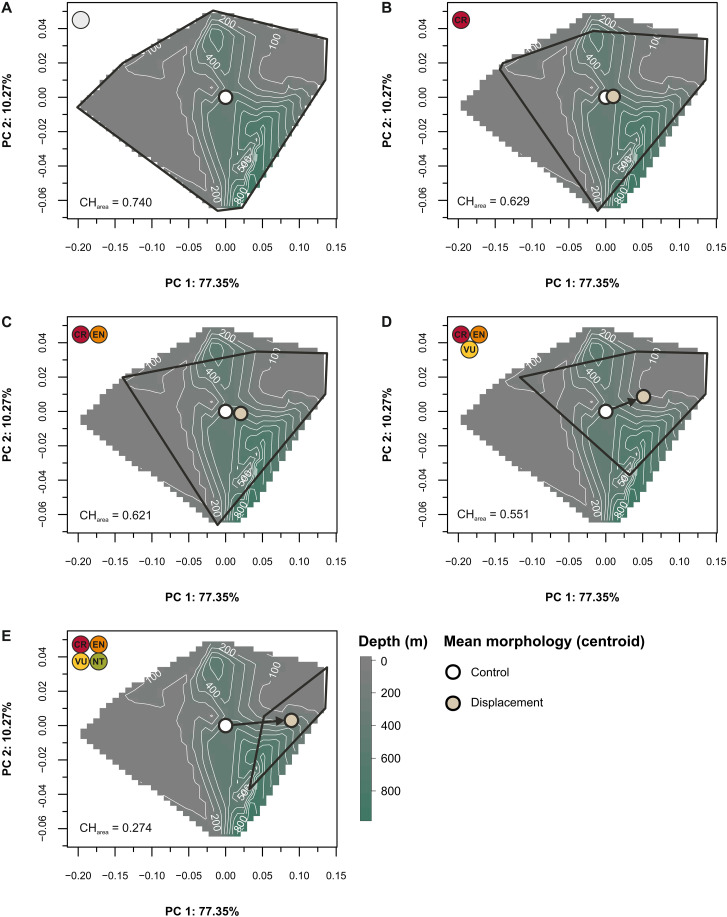
Erosion of dental morphospace. Extinction scenarios simulated by sequentially removing species by threat status in the ordinated space. (**A** to **E**) Convex hulls (black) enclose the species morphospace, illustrating its contraction with extinction. Open circles denote the mean morphology along PC1-PC2 for baseline (white) and extinction scenarios (beige), with arrows indicating the direction and magnitude of change. The raster grid represents interpolated depth (bathymetry in meters) values. CH_area_, generalized area of the hull. IUCN abbreviations: CR, Critically Endangered; EN, Endangered; VU, Vulnerable; NT, Near Threatened; LC, Least Concern.

**Fig. 3.**
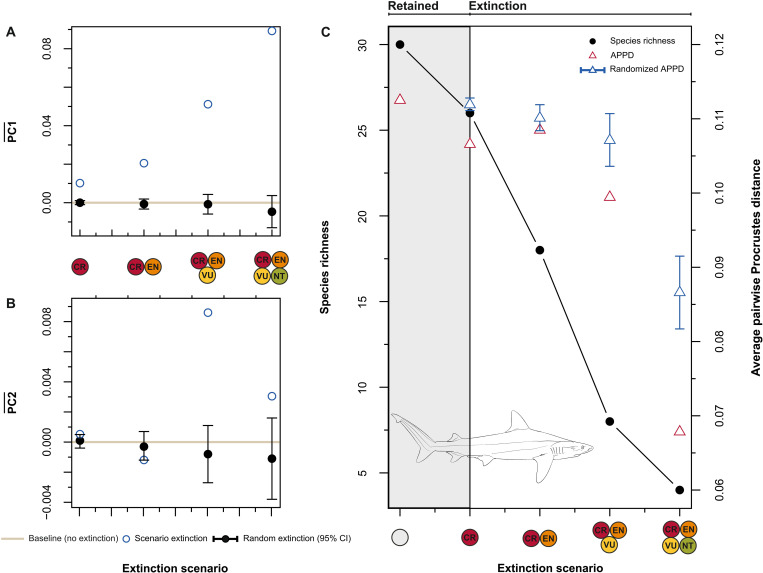
Extinction outcomes under current conservation threats versus random processes. Baseline mean values (solid horizontal line) compared against shifts in mean morphology along PC1 (**A**) and PC2 (**B**) under progressive species extinction (blue points) and random scenarios (black points). (**C**) The graph shows the dental disparity for 30 of 35 *Carcharhinus* species and how it would change in response to targeted (red triangle) and random (blue triangle) extinction. Randomized APPD values are shown along with 95% confidence intervals (CIs). Line drawing by Y. Arshad and inspired by illustrations in *Sharks of the World: A Complete Guide*, from chapter “CARCHARHINIFORMES: Ground Sharks” p. 520 *(Carcharhinus acronotus)* (*49*).

The extinction of Critically Endangered species would result in a 15% loss of occupied area as defined by the two main axes of variation (Fig. 2, A and B). Under this scenario, the reduction in shape space would affect peripheral morphologies loading negatively on PC1 and positively on PC2 (corresponding mostly to the loss of shallow-water and one deep-water species). The extinction of both Critically Endangered and Endangered species results in a 16% area loss, which, while not a substantial change, is accompanied by a shift in mean morphology and a lowered kurtosis, especially along PC1 (Fig. 2C). The third extinction scenario (Critically Endangered, Endangered, and Vulnerable) would reduce morphospace occupation by 25.5% (Fig. 2D). Such a scenario would cause vast depletion of tooth morphologies loading negatively on PC1 and extreme values on PC2 but also produce a shift in mean morphology compared to the baseline mean. Last, the loss of Near-Threatened species alongside the previous categories would vastly deplete the morphospace with a ~63% loss in areas occupied. This extinction scenario, while the least probable, would push the mean morphology toward positive PC scores on the first axis and leave a small pocket of morphospace intact inhabited by Least Concern species with a depth distribution of 20 to 286 m (Fig. 2E). Last, predicted outcomes under all threat scenarios examined deviate from expectations under a random extinction model, exceeding the 95% confidence interval along PC1, with smaller deviations observed along PC2 that are statistically significant for the two most intense extinction scenarios (Fig. 3, A and B).

### Morphological homogenization

We tested how morphological disparity erodes progressively with extinction severity on the basis of threat-based extinction scenarios and compared them to expectations under a null model of random extinction. The average pairwise Procrustes distance (APPD) will decrease under an extinction scenario where Critically Endangered species go extinct; this decrease is significantly larger than would be expected under random extinction of the same intensity (Fig. 3C). Conversely, as extinction severity increases to include Endangered species, APPD values converge back toward those expected under random extinction (Fig. 3C). As extinction progresses between the third and fourth extinction scenarios, the predicted decrease in APPD becomes more pronounced and remains more extreme than would be expected under scenarios involving the random losses of the same number of species. Overall, even random extinction tends to reduce APPD, but realistic extinction scenarios based on current threat levels would reduce it significantly further, accelerating morphological homogenization.

### Ecological diversity

**Fig. 4.**
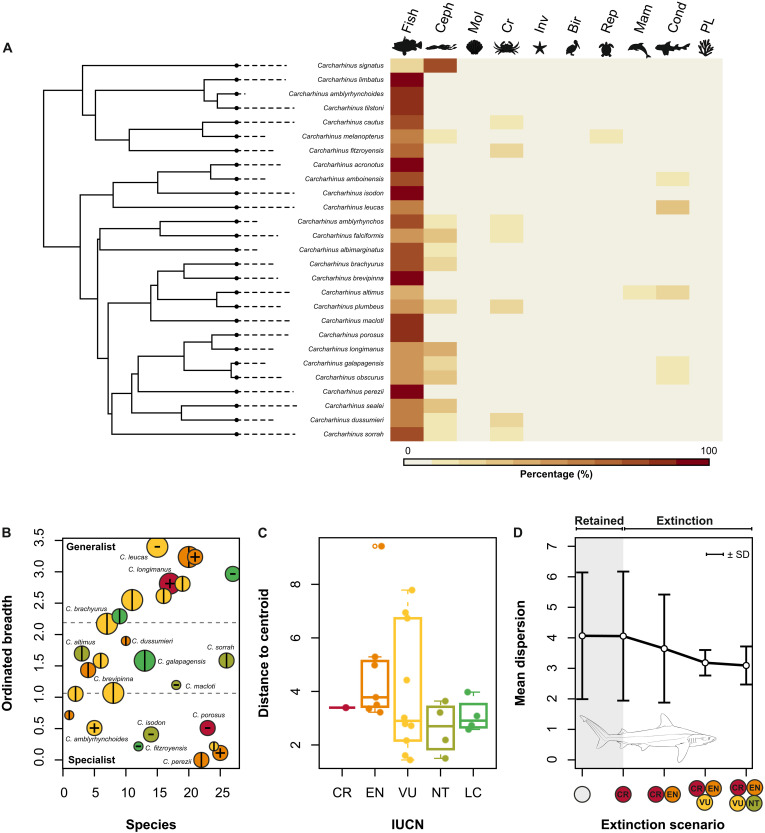
Ecological analyses. (**A**) Phylogenetic heatmap of diet compositions in 27 *Carcharhinus* species. (**B**) Scatterplot showing the ordinated dietary niche breadth for species ranging from “specialist” (low ODBs) to “generalist” (high ODBs) feeding ecologies. The dotted lines represent 33 and 66 percentiles, and the points are scaled relative to the categorical designations of small (<150 cm)–, medium (150 to 300 cm)–, and large (>300 cm)–bodied species [sensu Dulvy *et al.* (*18*)]. (**C**) Variation in ecological dispersion across IUCN categories, visualized with jittered box plots. (**D**) Calculated mean dispersion along with standard deviations under threat-based extinction scenarios. Line drawing by Y. Arshad and inspired by illustrations in *Sharks of the World: A Complete Guide*, from chapter “CARCHARHINIFORMES: Ground Sharks” p. 520 *(Carcharhinus acronotus)* (*49*). See the Supplementary Materials for the explanation of abbreviated prey items.

To explore the relationship between extinction threat, diet selection, and feeding specialization, we first examined the phylogenetic heatmap of species diets (Fig. 4A). The predominant dietary selection in *Carcharhinus* species is teleost fish, constituting ≥50% of the stomach contents in 25 of 27 species (Fig. 4A). The feeding on cartilaginous fishes, although present in the diets of many species, is less common than feeding on teleosts. Some species (e.g., *C. longimanus*, *C. falciformis*, *C. obscurus*, and *C. signatus*) have moderate to high proportional contribution of cephalopod prey. Diets incorporating at least 20% decapod crustaceans (crustacivory) occur in three species: *C. dussumieri*, *C. fitzroyensis*, and *C. plumbeus*. Feeding on tetrapods (birds, reptiles, and mammals) is negligible across *Carcharhinus* species with only two species showing high proportions of mammals (*C. altimus*) and reptiles (*C. melanopterus*) in their diets. Plants are rare in the diets of *Carcharhinus* species (~2% in *C. leucas* and 4.3% in *C. melanopterus*) (Fig. 4A).

Next, we looked at the relationship of ordinated dietary breadth (ODB) with threat status, habitat, body-size class, and morphology (Fig. 4B and fig. S3). We found no statistically significant relationship between ODB (i.e., dietary specialism/generalism) and threat status (*F*_4,22_ = 0.21, *P* = 0.93) or between ODB and habitat association (*F*_2,24_ = 0.096, *P* = 0.909) (Fig. 4B). There is a significant relationship between ODB and body-size class (*F*_2,24_ = 3.913, *P* = 0.03). Larger species gravitate toward more generalist feeding habits, medium-sized species are both specialist and generalist, and small-sized species exhibit specialized to intermediate diets (Fig. 4B). Linear regression of ODB against morphology (i.e., PC axes) shows a significant negative association with PC2 but not with PC1 (fig. S3). This correlates generalist diets (high ODB values) with broad and triangular teeth (negative PC2 scores) versus specialist diets with oblique-cusped teeth (positive PC scores).

To quantify the variance in feeding ecology across IUCN categories, we performed a multivariate homogeneity-of-group dispersion analysis (Fig. 4C). Although visual inspection of box-and-whisker plots (Fig. 4C) revealed large departures from the median in the Vulnerable category, we did not find statistically significant differences in dispersion patterns across IUCN categories (*F*_4,22_ = 0.98, *R*^2^ = 0.15, *P* = 0.42). Last, to assess how the loss of species under various extinction scenarios would affect the overall dietary diversity (i.e., ecological dispersion), we computed the mean distance to the centroid for each data subset corresponding to the previously described extinction scenarios (Fig. 4D). The effect of Critically Endangered species going extinct on the ecological dispersion is negligible. There is a decrease in dispersion once Endangered species are added to the extinction pool. Dietary homogenization becomes significant once Vulnerable and Near-Threatened species go extinct, with substantially reduced standard deviations (Fig. 4D).

## DISCUSSION

### Does selective extinction in shark promote phenotypic homogenization?

Quantifying the trophic effects of species extinction is challenging and compounded by variation in available information on their reproduction and life cycle, spatial distribution, and ecology. This challenge affects the species-rich, albeit morphologically conservative, genus *Carcharhinus* (*49*). For example, congeners classified as Critically Endangered like *C. obsoletus*, *C. borneensis*, and *C. hemiodon* have protracted lapses in occurrences (*54*, *64*, *65*) and, given their rarity, may be functionally extinct (*18*, *20*). In instances where little is known about a species, including whether it remains extant, it becomes difficult to interpret its ecological role or to understand the implications of its potential extinction. Our morphological analyses show that *C. borneensis* and *C. hemiodon* (both mesopredators of the “*Carcharhinus porosus* group”, sensu Garrick) occupy the periphery of the dental morphospace, and while their precise statuses remain nebulous, the extinction of these species would reduce the morphospace occupation of *Carcharhinus* sharks (Figs. 1 and 2). The extinction of *C. longimanus* (a species that occupies extreme values on PC2) would exert only a modest influence on the range of existing dental configurations. Yet, as a large, open-ocean, apex predator, the extinction of *C. longimanus* would have pronounced effects on food-web dynamics (*66*). This species is one of the few high trophic-level species within this genus, and among the minority that exhibits an epipelagic lifestyle—sharing this trait with the Endangered *C. signatus* and *C. plumbeus*, as well as the Vulnerable *C. amblyrhynchoides* and *C. falciformis* (the latter of which is also reef-associated) (*49*).

Using the dental archive of 30 *Carcharhinus* species, we assessed the impact of extinction scenarios on shape-space occupation. We find that the sequential loss of species based on IUCN status will reduce areas of morphospace in a nonrandom fashion. The extinction of all threatened species (i.e., Critically Endangered, Endangered, and Vulnerable) would result in a loss of nearly 25.5% of morphospace (Fig. 2D); disproportionately affecting oblique and blade-like tooth structures. Furthermore, the extinction of Critically Endangered species would increase the similarity in tooth structures among surviving species. Conversely, the combined extinction of Critically Endangered and Endangered species (40% species loss) would not decrease morphological disparity much further (i.e., compared to the baseline level) owing in large part to redundancy in morphospace (Figs. 1 and 2). The largest change to morphospace occupation is associated with the loss of all threatened species, which would vacate large areas, especially along PC1 (Fig. 2, D and E). This last scenario would cause a decline in dental disparity compared to the baseline level (this decline is also stronger than would be expected under random extinction at a comparable intensity; Fig. 3C).

Because threatened species make up 73.3% of the dataset, the scale of loss caused by either random or targeted extinction (or a combination of both) would be severe enough to cause a decline in dental disparity (this signal persists when extended to the analysis of intraspecific variation; fig. S4, A and B). This convergence of outcomes suggests that beyond a certain threshold, even selective survival would not maintain enough phenotypic variability. Surviving species would necessarily persist in a substantially altered ecospace (Fig. 2E). Overall, nonthreatened species (e.g., *C. cautus* and *C. fitzroyensis*) are largely confined to positive areas along our main axis of variation (also see figs. S5 and S6 for an intraspecific overview of morphospace). However, global conservation designations may not apply to specific regions as exemplified by *C. galapagensis*, a species that is locally extirpated in the Archipelago of Saint Paul’s Rocks (*67*). Moreover, IUCN status designations are a snapshot of threat assessment and not intended as a predicted extinction sequence per se. Instead, our results provide a glimpse into how future extinction could transform the diversity of tooth morphology and the ecological role associated with them.

### Ecological consequences

Among the *Carcharhinus* species designated as Endangered and Vulnerable, 14 are associated with coral reef environments (Fig. 1). This situation is consistent with previous research showing that 59% of coral reef–associated shark and ray species are at high risk of extinction (*51*). More broadly, sharks and rays fill many ecological niches in reef habitats (*68*–*71*), which makes them functionally important trophic components of reef ecosystems (*51*). Our analyses of dietary niches reveal that large-bodied species tend to be more generalist in their feeding habits (Fig. 4B). However, there is no strong evidence linking dietary niche to preferred habitats, including reefs. Furthermore, a comparison across the IUCN categories did not support vast heterogeneity in *Carcharhinus* shark diets. We cautiously interpret these results, recognizing that while many *Carcharhinus* species are piscivorous, they exhibit considerable variation in prey preference, feeding mechanics, and spatial-temporal hunting strategies (*50*, *71*–*74*). Accordingly, the gut-content diet classifications (*75*) used here provide a good overview of broader feeding involving main prey items but cannot be used to determine specialization on select prey. Using the stomach content data, we nonetheless show that extinction of threatened species would affect the general prey base, which becomes more focused on fish and crustaceans under the more intense extinction scenarios (Fig. 4D). Feeding on nonfish prey would therefore decline as threatened species go extinct (e.g., moderate reptile consumption in *C. melanopterus* and the high reliance on squids in *C. signatus*) and could theoretically spark greater competition among species and potentially homogenize ecological roles. However, our global approach and the emphasis on one genus of sharks are not sufficient to generalize about the wider effect on disparate ecosystems. Moreover, sympatric species with similar lifestyles and shared traits (e.g., Australia’s Blacktip Shark complex) (*76*) could potentially buffer against the effects of species extinction.

### Macroevolutionary implications

The loss of species richness is anticipated to homogenize the functional diversity of many animal communities (*31*, *77*–*80*). However, its impact on morphological diversity has received comparably less attention (*31*). Studies of mass extinction events across geologic time often reveal incongruent patterns in how various facets of biodiversity are affected (*4*). This finding presents a challenge in predicting how the morphological diversity of modern biotas will be shaped by analogous outcomes generated by human influence. Sharks offer a unique opportunity for knowledge transfer across temporal scales as they preserve an abundant record of teeth for extant and fossil species (*38*, *39*, *81*). Historically, the study of shark teeth has been motivated by a desire to resolve differences in alpha taxonomy. This situation is well encapsulated by remarks made by J. A. F. Garrick in his seminal monograph on *Carcharhinus* species:

“If one takes an overview of shark systematics in general, the best single feature that could be cited for determining similarities and differences between taxa at all levels, but perhaps predominately at the generic level, is the shape of the teeth” [(*37*), p. 20].

It is, however, the morphological and ecological characteristics that determine an organism’s fitness or performance (*82*, *83*). So far, the analytical treatment of shark teeth for the purpose of understanding the evolutionary and ecological consequences of extinction has been the purview of paleobiology (*34*, *42*–*44*, *63*, *84*). Conversely, neontological work has developed workflows to classify species (*38*) along with the quantification of heterodonty between species (*41*, *85*) and among species populations (*86*). This evidence shows that tooth shape, while only one aspect of a species phenotype, can provide important insights into ecological function (*40*) and morphological diversity (*34*, *43*, *44*).

Applications to the fossil record of shark tooth morphology have recovered a series of complex patterns in the morphospace, including the selective demise of morphotypes associated with large-bodied specialized species at the end-Cretaceous mass extinction event (*43*). Such analysis has also recovered evidence of postextinction proliferation of an array of tooth shapes associated with generalist lifestyles (*34*, *43*, *44*). The specific threat of a selective decline in many elasmobranch species today is starting to bear resemblance to one of the worst mass extinction episodes in Earth’s history. Using a geometric morphometric approach, we show how extinction outcomes based on IUCN status would affect the morphological diversity of *Carcharhinus* species—a group of sharks that face excessive pressure from overfishing (*55*). Our analysis indicates that the range of dental structures that have evolved over millions of years risks being lost, leaving increasingly homogenized shark dentitions ill-suited to diverse ecological roles.

## MATERIALS AND METHODS

### Experimental design

#### Data sources

A total of 1256 complete tooth crowns (from upper and lower jaws) representing 30 species was compiled from photographed dentitions derived from refs. (*50*, *87*). Unless stated otherwise, the teeth represent positions along the functional row and correspond to the right side of each jaw. The resulting dataset includes information drawn primarily from a single individual per species and from two individuals in 14 species (figs. S7 and S8). The sample sizes ranged from 14 to 69 individual teeth per species (with a mean of 42 teeth per species). We opted to exclude images of teeth that either showed signs of deformation or extensive breakage or whose apex point could not be differentiated (the latter only applied to very posterior teeth). The numbering of tooth positions follows the natural sequence from the symphysis at the front of the mouth toward the commissure at the back of the jaw. Before landmark digitization, we digitally cropped individual teeth from the photographed dentitions and standardized the image orientation to facilitate a direct comparison. This standard orientation included presenting each tooth in labial view and was digitally rotated so that the apex of each tooth was facing up (fig. S9).

We prioritized photographed dentitions with scale bars and associated specimen data (i.e., total length, sex, and locality); only one species, *C. tilstoni*, lacked body-size information. The nomenclature and taxonomic assignments in (*50*, *87*) were mostly taken at face value but also cross-referenced against FishBase (*88*), Eschmeyer’s Catalog of Fishes Online Database (*89*), and other primary literature cited herein (*49*). Several species were excluded from our analysis because of lack of an accessible photographed dentition, lack of IUCN threat data, and/or taxonomic questions regarding the validity of a particular species. For example, although we maintain a neutral stance on the validity of *C. wheeleri*, some studies (*61*, *90*) consider this species a junior synonym of *C. amblyrhynchos*, and threat data for the former are not provided by the IUCN. Our sampling strategy captures only limited intraspecific variation in dentition across all sampled shark species. In addition, our approach does not account for sexual dental dimorphism, dental asymmetry, regional dental differences, and other more subtle forms of heterodonty (i.e., gynandric heterodonty) (*38*, *81*). Accounting for these types of heterodonty would require exceptionally large datasets for each taxon (*91*) and is beyond the scope of the present study. It is our view, however, that our current sampling strategy is sufficient to test our hypotheses in a broader sense.

The conservation status (Critically Endangered, Endangered, Vulnerable, Near Threatened, and Least Concern) for each species were derived from the IUCN (www.iucnredlist.org/). Although IUCN statuses can vary at the regional and national levels (*92*), herein, we used the global conservation status for each species because of the widespread biogeographic range of this genus (*49*). Evidence from the fossil record demonstrates that geographic range size is among the strongest correlates of extinction probability through deep time [e.g., (*93*)]. This provides, in our opinion, support for using the IUCN as a predictor of extinction risk, especially as modern threat classification relies on geographic range as one of its main criteria (*94*). We also compiled trait data for each species (categorical and numeric variables) from ref. (*18*) including maximum depth limit (in meters), body-size class (small, medium, and large), and habitat (coastal and pelagic). A separate habitat category emphasizing reef-associated versus non–reef-associated species was used following ref. (*95*) and complemented using habitat summaries listed in (*49*). Last, we compiled gut content–derived diets, expressed as proportional compositions, for 27 species from ref. (*75*). All prey categories (table S1) were considered except for zooplankton (mainly euphausids), which were removed because they contributed 0% in all species (fig. S10). Before analysis, we applied the centered log-ratio transformation to our diet data using the compositions version 2.0-8 R package (*96*).

#### Geometric morphometrics

We used two-dimensional landmark-based geometric morphometrics to quantify variation in tooth crown shapes of *Carcharhinus* species. Our digitization scheme (fig. S9) included (i) three fixed points corresponding to the crown-root junction and the apex and (ii) two curves with semilandmarks (157 points) traced on the mesial and distal sides of the crown (*34*, *42*–*44*). All digitization was achieved using TpsDig software version 2.32 (*97*) and was postprocessed in R using custom code to ensure the point resampling and equidistance of curves. The raw landmark data were subsequently passed on to a generalized Procrustes superimposition analysis (*98*, *99*) using the gpagen function from the geomorph version 4.0.8 R package (*100*). The positions of sliding semilandmarks were determined on the basis of a bending energy criterion estimated via approximate thin-plate spline mapping (*100*).

### Statistical analysis

#### Shape analysis

Ordination via a principal components analysis was performed on the Procrustes-aligned coordinates using the gm.prcomp function in geomorph. Our main analysis was performed using the mean configuration of species. To explore tooth variation caused by dignathic heterodonty, we proceeded to compute species level mean configurations on the basis of upper and lower jaw teeth and subjected these to separate ordinations. Our species-level analyses involved the projection of a tree topology onto the shape space, leveraging a maximum clade credibility tree computed from a tree distribution of cartilaginous fishes (*101*). We subsequently mapped our categorical data variables (IUCN status, body-size class, and habitat) onto the phylomorphospace. Visualization of shape variation along the extreme values of the PC axes was constructed using thin-plate spline transformation grids (*102*).

Morphological differentiation in the shape space among species assigned to the various IUCN categories was evaluated via a one-way analysis of variance (ANOVA) design using a randomized residual permutation procedure (RRPP) for significance testing at an α level of 0.05 (*103*). Pairwise comparisons between group means were performed using the pairwise function from the RRPP version 2.0.3 R package (*103*). Collectively, our statistical tests used the full data space (i.e., all dimensions) and were not limited to single axes of variation. Last, we assessed the phylogenetic signal and related phylogenetic signal effect size in tooth shapes across *Carcharhinus* species. This was quantified using the geomorph R functions physignal and physignal.z (*100*).

#### Intraspecific variation

Equivalent morphospace analysis was performed without reliance on species averages but, instead, using the full dataset (figs. S5 and S6). To test whether species are different in the shape space, we used an ANCOVA (analysis of covariance) design with fixed effects including log-transformed centroid size, species identity and its interaction with centroid size, and dignathic heterodonty (table S2). Furthermore, we fitted an ANOVA to test whether differences in morphospace occupation among IUCN classes persisted when using the full specimen-level dataset (table S3). For exploratory purposes, we also constructed morphospace visualizations for *Carcharhinus* species on the basis of tooth positions from the upper and lower jaws (fig. S11, A and B).

#### Extinction scenarios

The erosion of morphospace was assessed by excluding species sequentially on the basis of IUCN status designations and by tracking shifts in the occupancy (area) and the displacement (magnitude and direction) of average morphology (i.e., centroid) along the two main axes of variation. We mapped bathymetry onto our shape space to account for how vertical habitat variation would be affected by such extinction scenarios. This allowed us to concomitantly assess whether future losses would disproportionately affect species associated with specific depth zones. To this end, we used bivariate interpolation to generate a smooth surface (i.e., counter map) of depth values overlaid onto our shape space using the interp function from the akima version 0.6-3.4 R package (*104*). We also performed a series of simulation-based extinction analyses on the basis of a Monte Carlo process whereby species were randomly removed at varying extinction intensities (4, 12, 22, and 26) using 100 replicates per extinction level. From this randomization exercise, we estimated an overall average along with 95% confidence intervals on the basis of the sample distributions corresponding to our main axes of variation.

A simulation procedure was also conducted (100 replicates per extinction level) to assess changes in morphological disparity under random extinction. We used the APPD (*105*–*107*) as our preferred measure of disparity and generated 95% confidence intervals around the mean for each extinction scenario. APPD can be defined as

$$\text{APPD}=\frac{2}{n\left(n-1\right)}\sum_{i=1}^{n}\sum_{j=i+1}^{n}d_{\mathrm{ij}}$$

where *n* is the number of specimens, and $d_{\mathrm{ij}}$ is the Procrustes distance between the landmark configurations of the ith and jth specimens. A higher APPD value indicates greater dissimilarity among specimens (=differentiation), whereas a lower value suggests that the specimens are more tightly clustered (=homogenization).

#### Variance-based analysis

We complemented our disparity analysis to account for within-species variation by estimating the Procrustes variance (defined as the trace of an *n* × *n* covariance matrix found from cross-products of observations) (*108*) using the morphol.disparity function from geomorph (*100*). As part of this analysis, we first computed the Procrustes variance for groups defined by the IUCN by summing the squared Procrustes distances of specimens within each group. Second, we evaluated how the exclusion of conservation groups would influence the overall morphological disparity in the entire dataset (fig. S3, A and B). Last, the disparity profile of *Carcharhinus* species was estimated for teeth originating from the upper and lower jaw (fig. S12). This served as a test of whether upper and lower teeth contribute differently to the total variance within a given species.

#### Niche breadth

Related to our morphological analyses, we asked whether species with specialist versus generalist diets are threatened with extinction more than other species. Using a binary (presence/absence) representation of our dietary matrix of 10 prey categories, we calculated the ODB for 27 species for which diets were available using the ordiBreadth version 1.0 R package (*109*). ODB is quantified as the sum of squared Euclidean distances of each used prey from the consumer’s centroid in the ordination space. The prey’s positions are derived by applying principal coordinate analysis to a dissimilarity matrix of prey-consumer associations (*109*). Using an ANOVA, we then tested whether species with different IUCN statuses, habitats, and body-size classes have different breadth values. The results from our ODB analysis were visualized using the raw ordinated breadth values (i.e., total breadth) plotted against the number of species. Each point representing a species was scaled to match body-size classes, color filled on the basis of IUCN categories, and overlaid with symbols corresponding to habitat associations. Last, to aid the interpretation of where points fall along the specialist-generalist continuum, we computed the 33rd and 66th percentiles using the quantile function from the stats version 4.4.1 R package (*110*).

#### Ecological diversity

The loss of distinctive traits in species could potentially affect the breadth of ecological diversity (i.e., feeding heterogeneity). Accordingly, we evaluated how various extinction outcomes could influence feeding ecology among *Carcharhinus* species. More specifically, we tested for a homogenization effect on the main prey types exploited among group members. We first calculated the Euclidean dissimilarity matrix on the centered log-ratio–transformed diets for all species and four reduced data subsets representing four extinction scenarios:

1) Critically Endangered.

2) Critically Endangered and Endangered.

3) Critically Endangered, Endangered, and Vulnerable.

4) Critically Endangered, Endangered, Vulnerable, and Near Threatened.

Next, we quantified the multivariate dispersal (i.e., variances) of species feeding ecology by IUCN status using the betadisper functions in the vegan version 2.6-6.1 R package (*111*). This was followed by a permutation test to evaluate statistical differences between group dispersion using the permutetest function, also from the vegan R package. Subsequent analyses were then performed using the scenario-based, dissimilarity matrices without specifying a grouping effect. Last, shifts in community spread following the sequential removal of species based on IUCN status were evaluated by computing the mean distance to the centroid among the remaining species.

#### Literate programming and reproducibility

All our analyses were performed in R version 4.5.0 (*110*), and we used the Quarto publishing system (*112*) to produce a browsable, self-contained HTML file with all code, tables, figures, and text. We also containerized our working environment to enable the persistent reproducibility of our analytical pipeline without obstruction caused by software dependence (*113*, *114*). This was achieved using the Docker platform (*113*), and we used a minimal Debian-based Docker image from the Rocker Project (https://rocker-project.org/images/) to ensure a reproducible and portable R environment for our analysis.
