# Supplementary material for: Extinction threatens to cause morphological and ecological homogenization in sharks
Source: Sci Adv. 2025 Oct 29;11(44):eaea0278. doi: 10.1126/sciadv.aea0278 (PMC12571088; doi:10.1126/sciadv.aea0278)
Supplement: Supplementary file 1 — Figs. S1 to S12 Tables S1 to S3 [file sciadv.aea0278_sm.pdf]

Science Advances
AAAS

Supplementary Materials for

Extinction threatens to cause morphological and ecological homogenization in sharks

Mohamad Bazzi *et al.*

Corresponding author: Mohamad Bazzi, bazzi@stanford.edu

Sci. Adv. **11**, eaea0278 (2025)
DOI: 10.1126/sciadv.aea0278

This PDF file includes:

Figs. S1 to S12
Tables S1 to S3

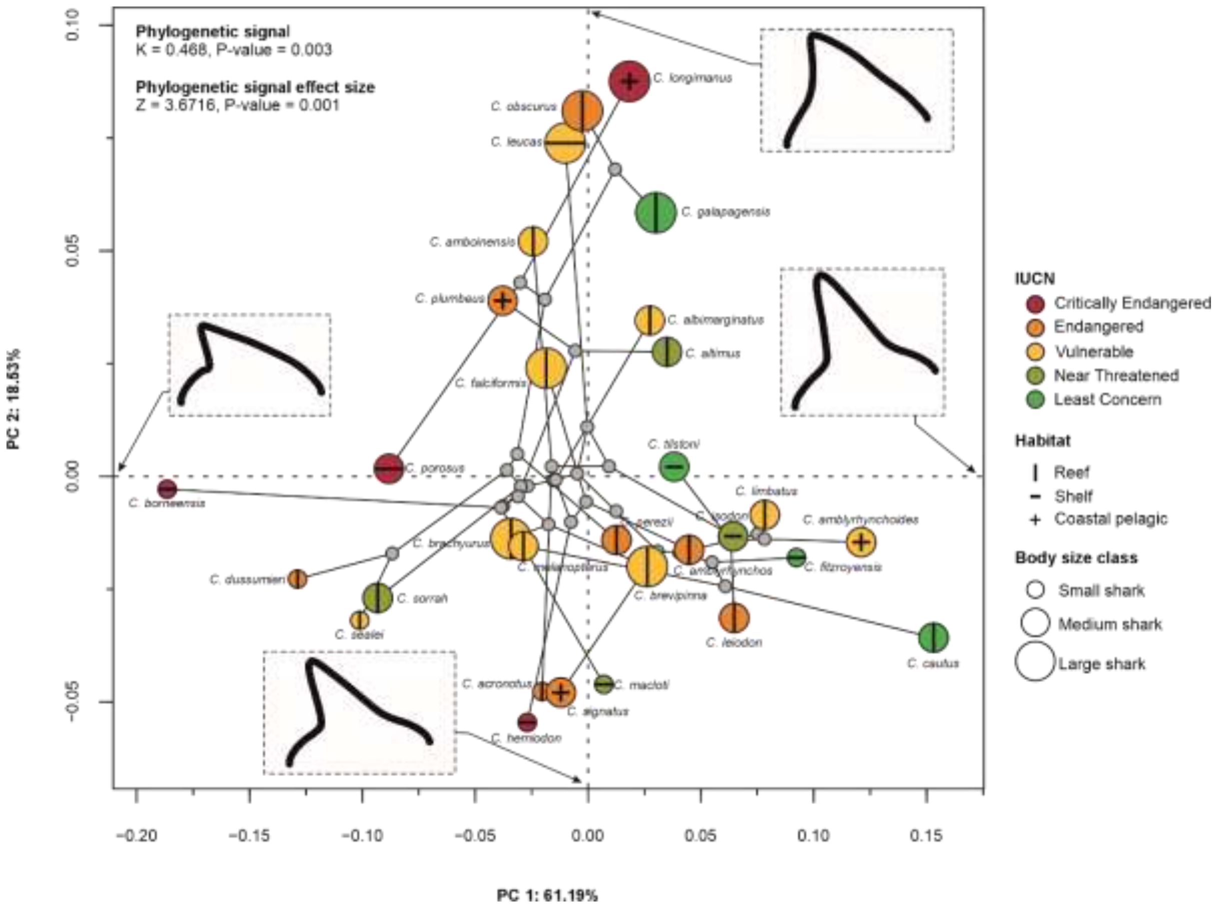

Fig. S1. Phylomorphospace of *Carcharhinus* species based on upper jaw teeth. Points are color-coded based on IUCN status and scaled relative to the categorical designations of small (<150 cm), medium (150–300 cm) and large (>300 cm) bodied species (*18*). Habitats are indicated by symbols (vertical bar, plus and minus signs). Thin plate spline deformation grids indicate theoretical shape at the extremes of PC1 and PC2. Phylogenetic signal and effect size estimates for Procrustes shape data based on upper jaw teeth are provided.

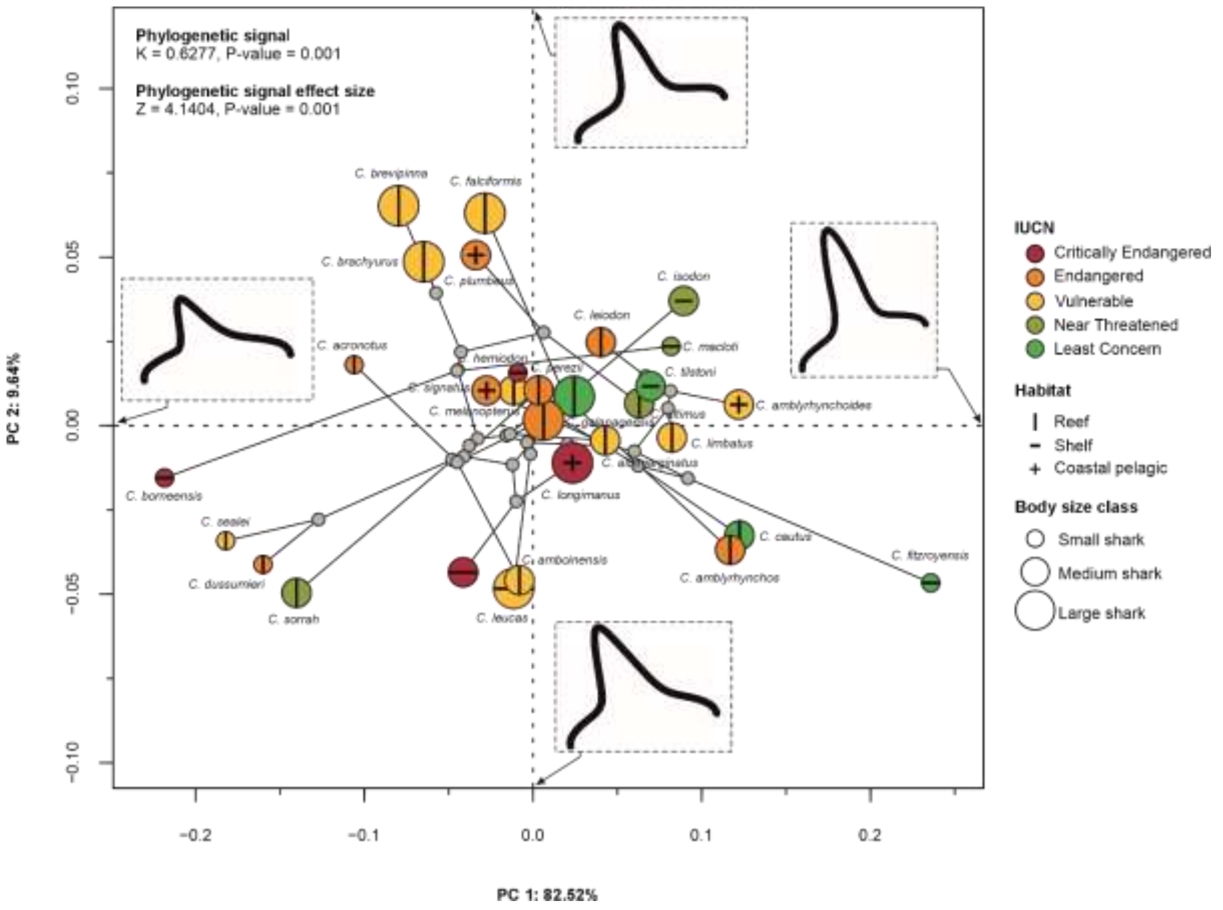

Fig. S2. Phylomorphospace of *Carcharhinus* species based on lower jaw teeth. Points are color-coded based on IUCN status and scaled relative to the categorical designations of small (<150 cm), medium (150–300 cm) and large (>300 cm) bodied species (*18*). Habitats are indicated by symbols (vertical bar, plus and minus signs). Thin plate spline deformation grids indicate theoretical shape at the extremes of PC1 and PC2. Phylogenetic signal and effect size estimates for Procrustes shape data based on lower jaw teeth are provided.

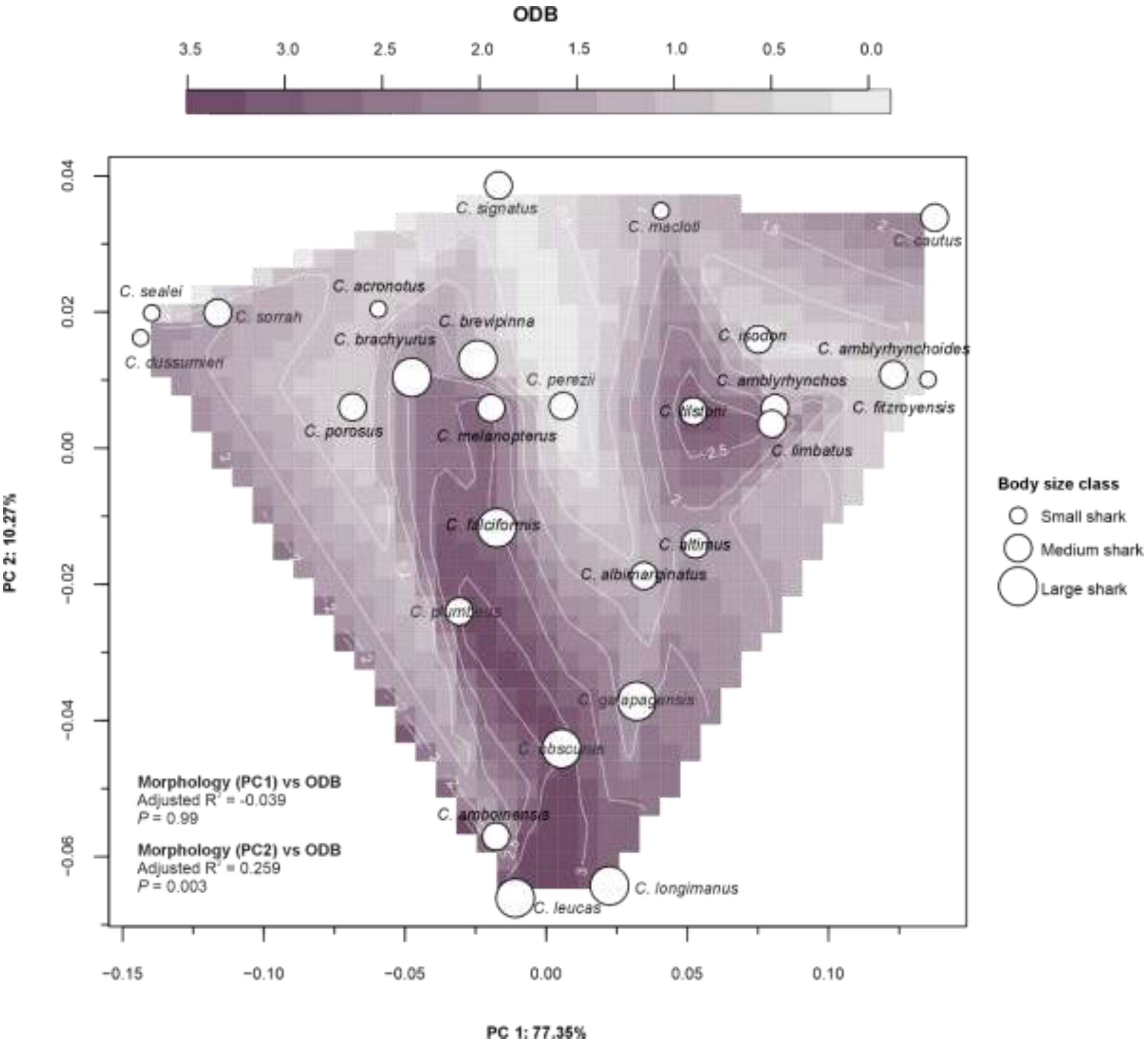

Fig. S3. Mapping ordinated niche breadth values onto dental morphospace. Bivariate graph showing the distribution in morphospace based on species averages along with an overlaid raster-grid of computed ordinated niche breadth for 27 species. Low ODB values indicate dietary specialization whereas high ODB values reflect dietary generalism. Linear regression results (adjusted R^2 and *P*-values) are reported for ODB versus PC1 and ODB versus PC2.

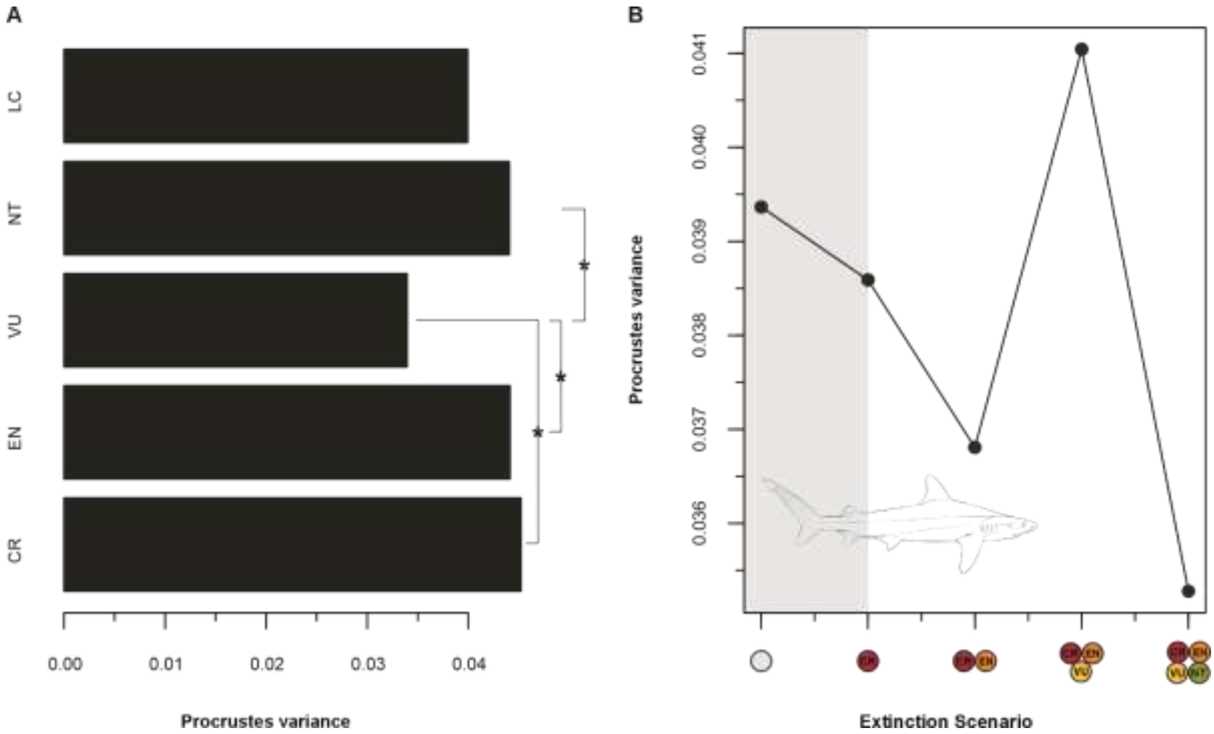

Fig. S4: Dental disparity estimated as Procrustes variance. (A) Bar plot illustrating disparity estimates derived from the full specimen-level dataset (n = 1256), with species observations grouped according to their assigned global IUCN status. Asterisks indicate statistical significance ($p << 0.05$) between groups evaluated using RRPP with 999 permutations. **(B)** The effect on dental disparity by progressively excluding *Carcharhinus* species by higher threat status. The results show how the removal of Critically Endangered (CR) and Endangered (EN) species will reduce the overall variance. Line-drawing by Yameen Arshad and inspired by illustrations in Shark of the World (*49*).

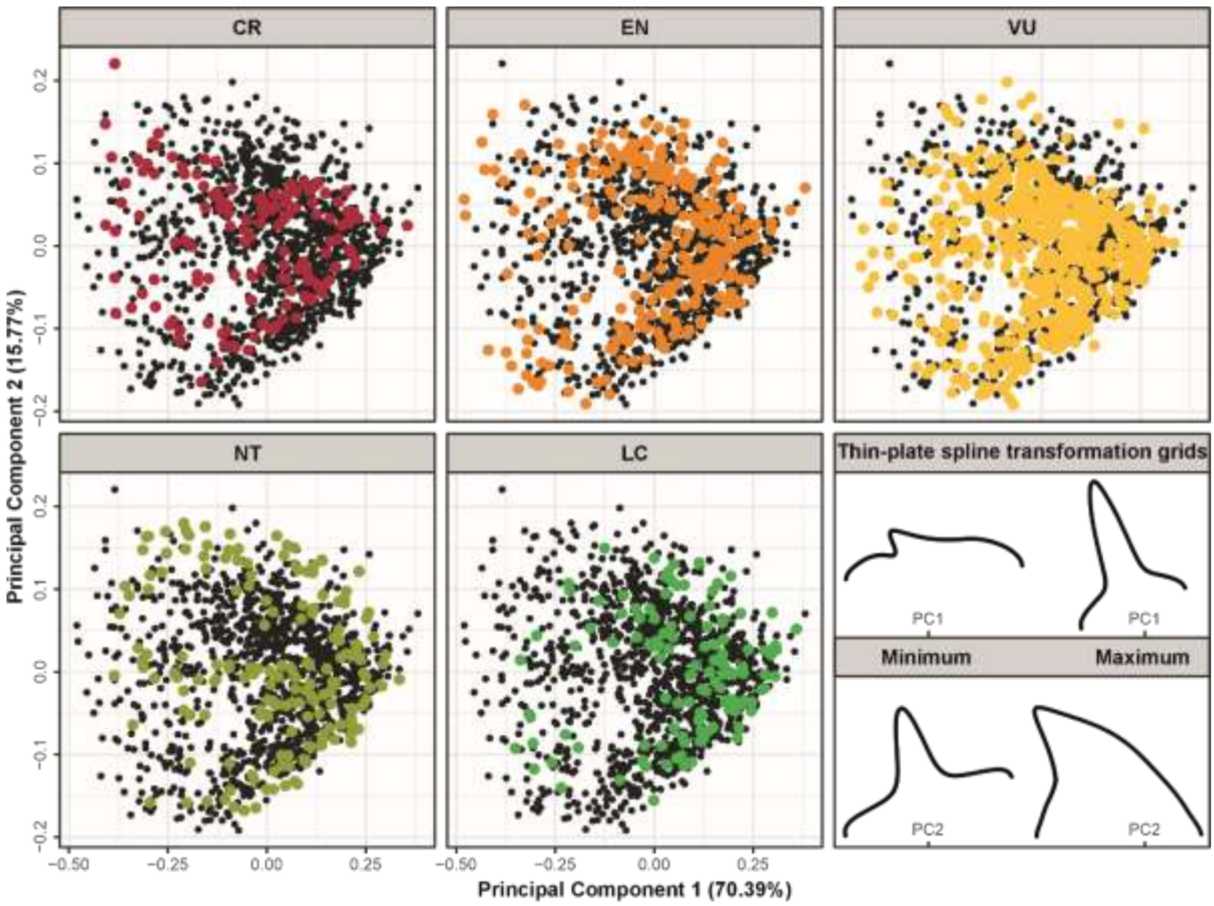

Fig. S5: IUCN status mapped onto the dental morphospace of *Carcharhinus*. Ordination plots show morphological variation from the GPA-aligned coordinate dataset. The panels highlight species observations (i.e., specimens) and are color-coded according to their designated IUCN threat category. IUCN abbreviations are explained in the main text.

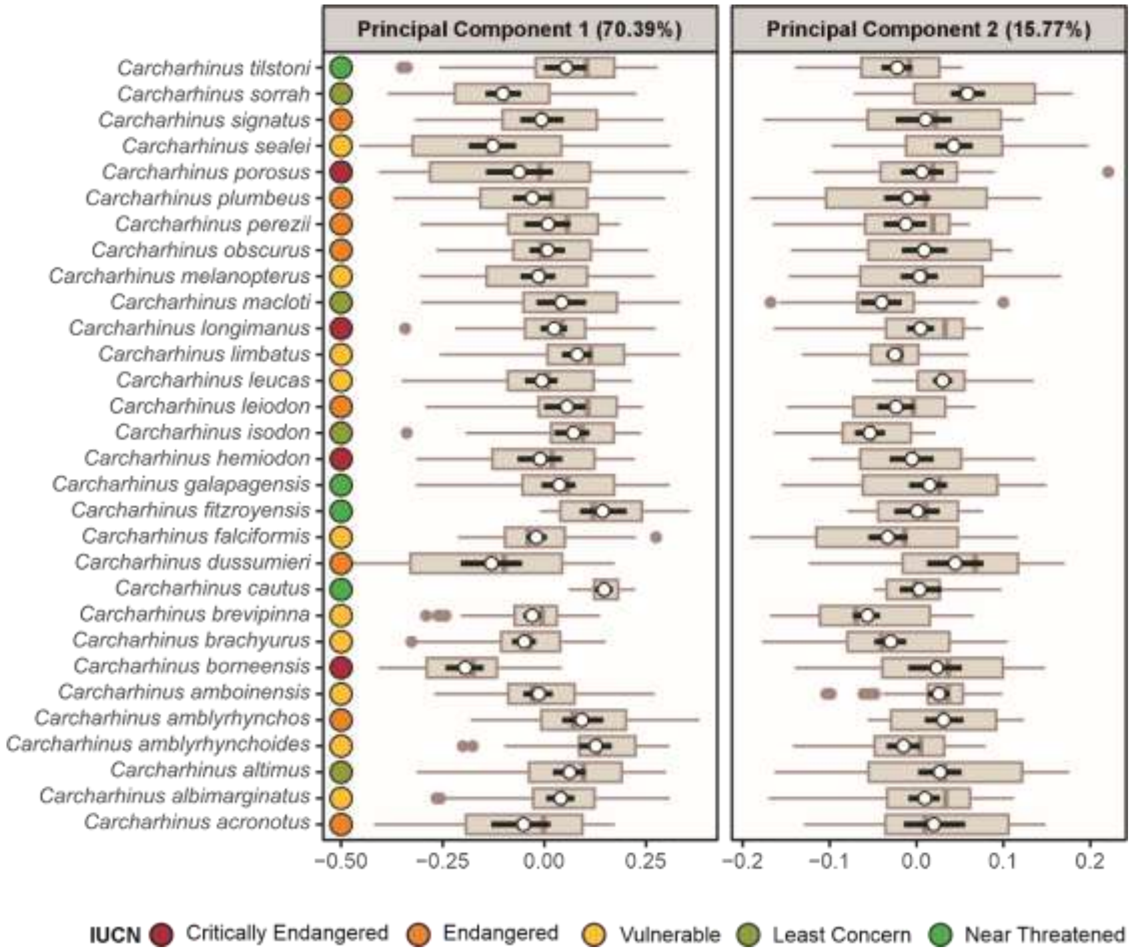

Fig. S6: Morphospace occupation of *Carcharhinus* species. (A–B) Box-and-whisker plots depicting morphological variation along PC1 and PC2. Summary statistics include the median, 25th and 75th percentile hinges, whiskers, and outliers. Each box also incorporates the arithmetic mean and estimated 95% confidence interval.

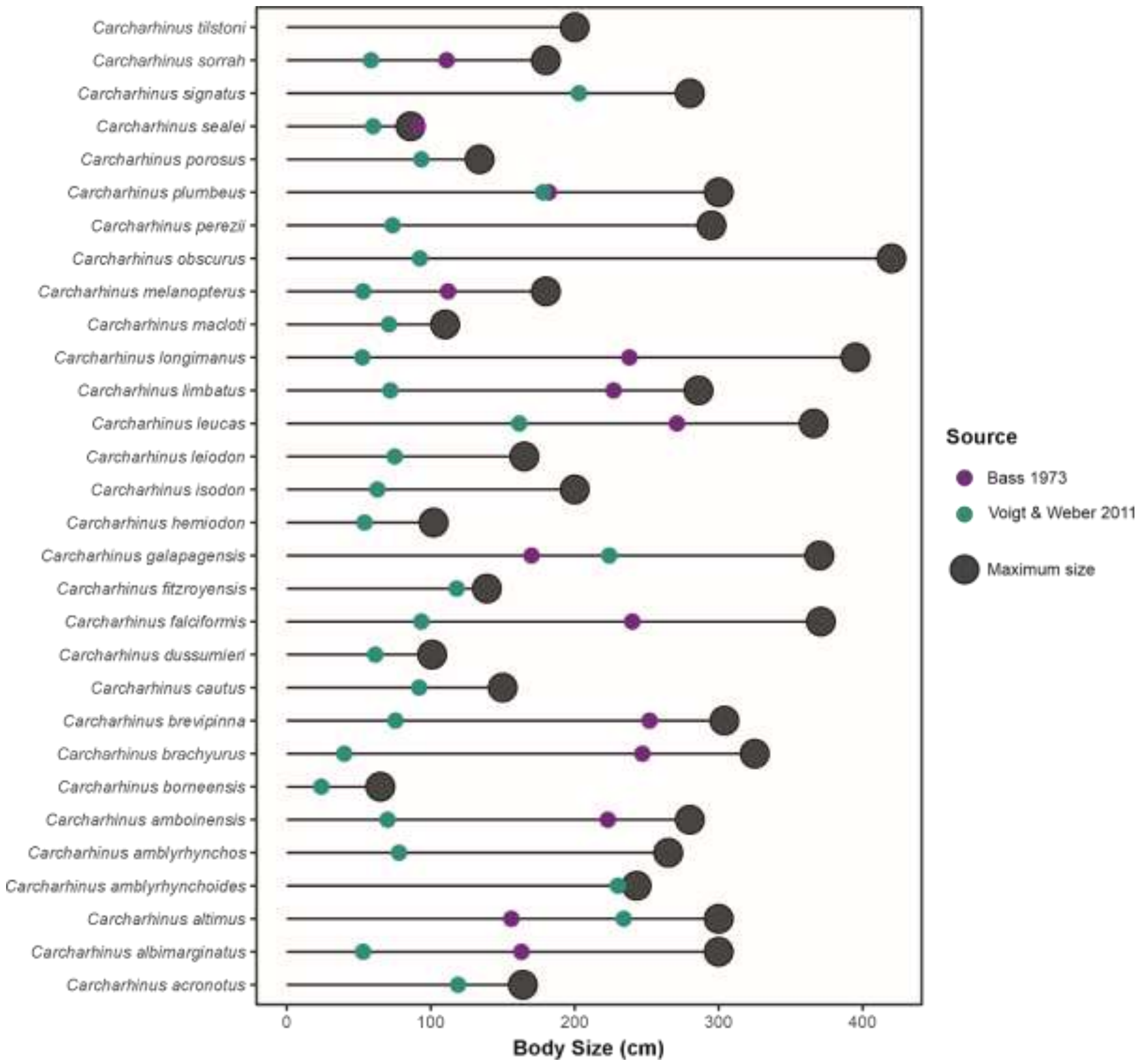

Fig. S7: Maximum potential body-size versus observed size. Body lengths of sampled individuals compared with published maximums from (*49*).

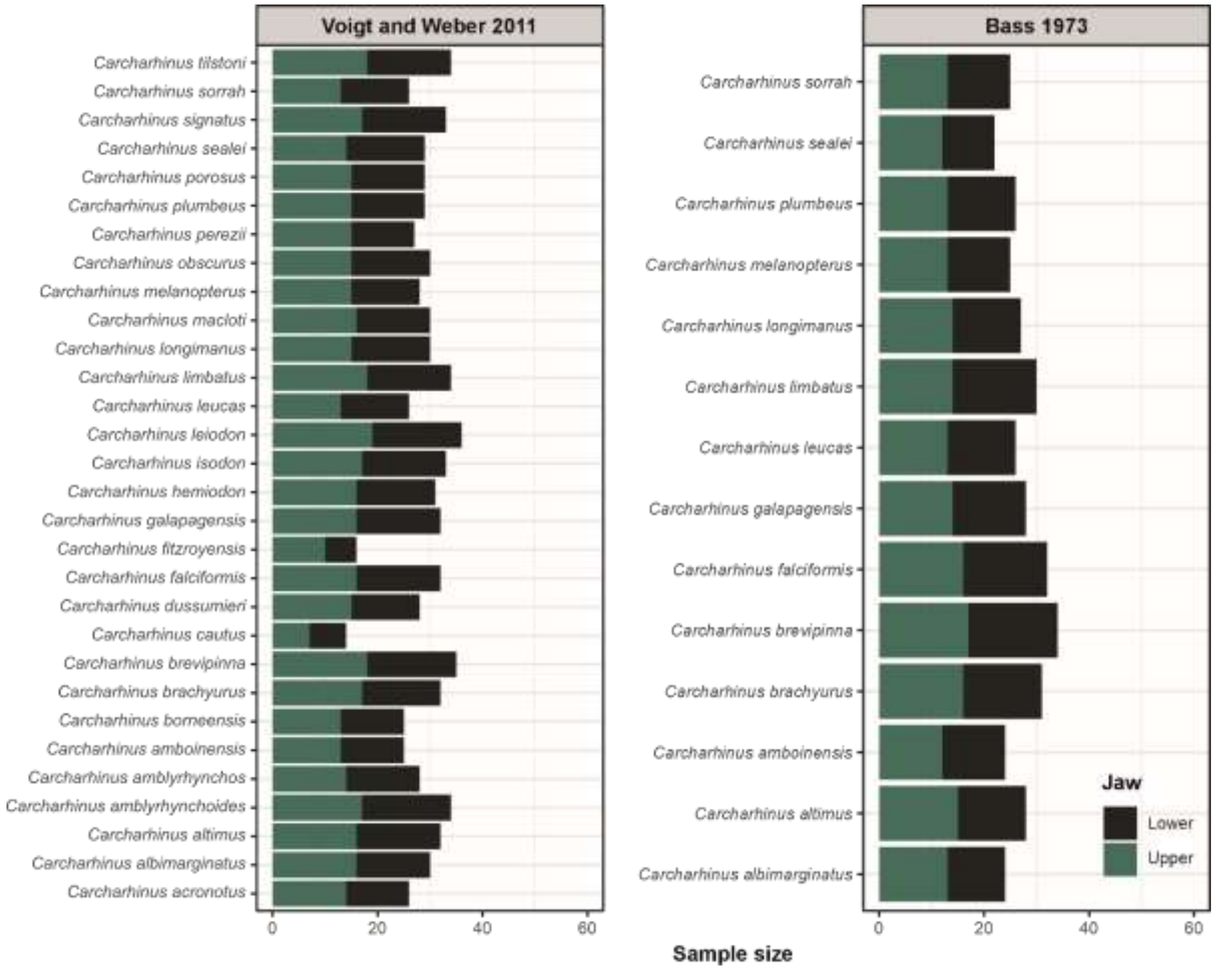

Fig. S8: Number of upper and lower teeth sampled per *Carcharhinus* species. We managed to sample 874 specimens from (*50*) and 382 from (*87*).

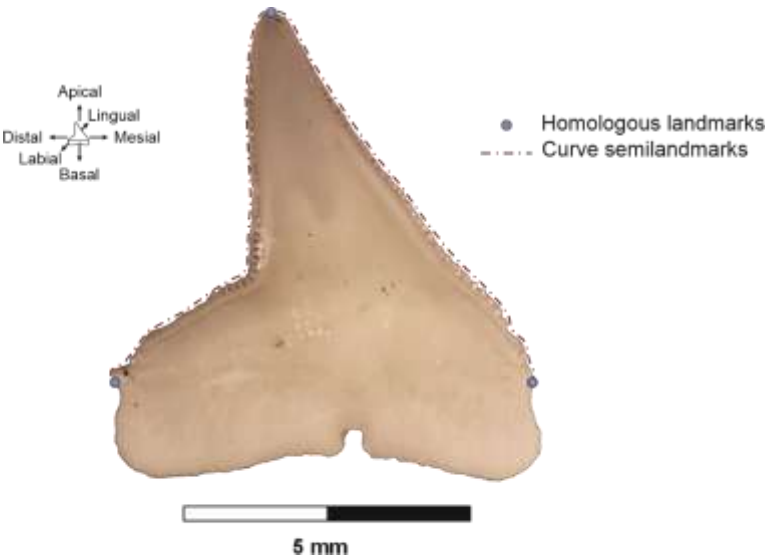

Fig. S9: Digitization scheme. Upper left anterolateral tooth from a mature female (total length = 1155 mm) *Carcharhinus acronotus* (MSC 51981) from the Gulf of Mexico.

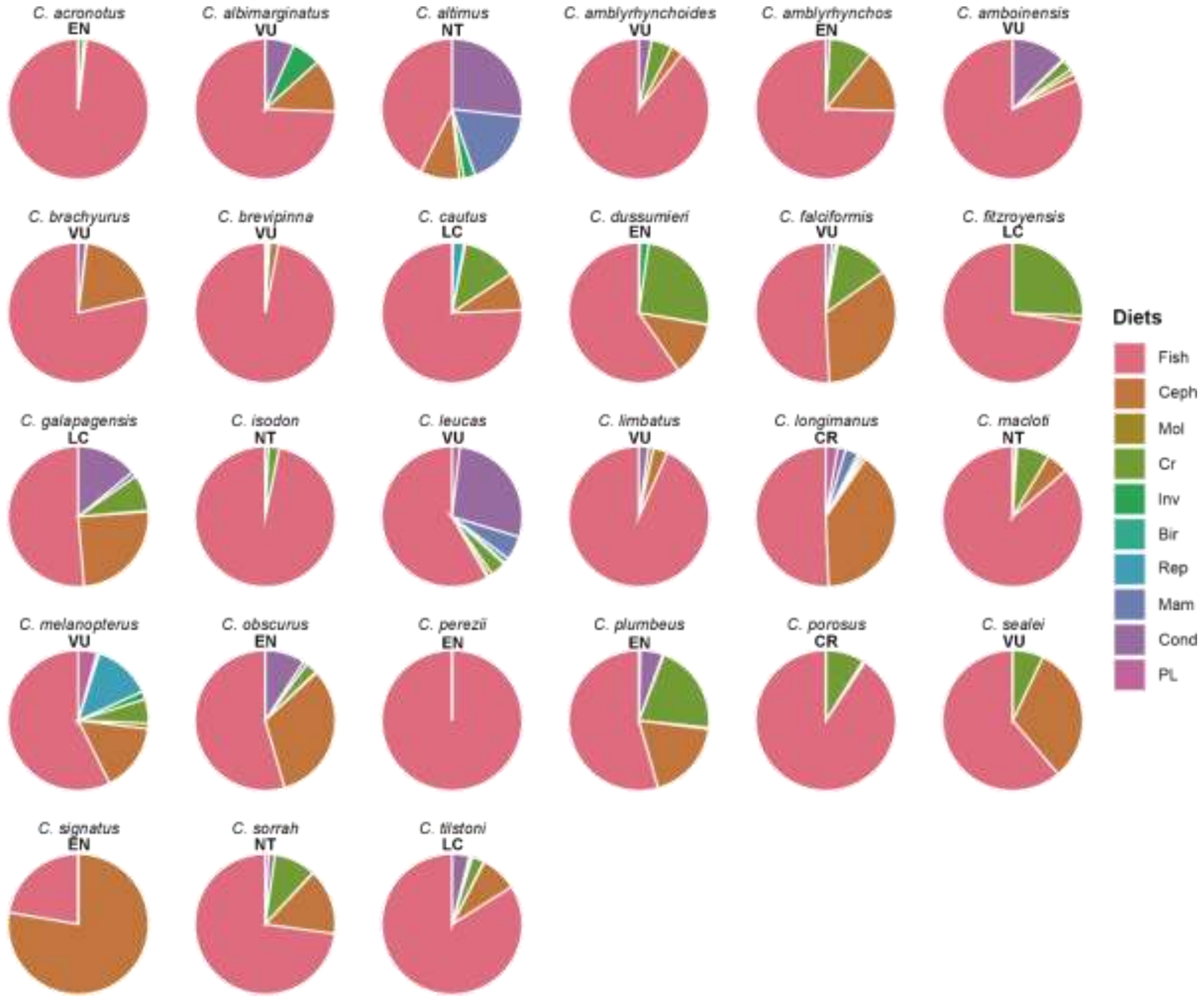

Fig. S10: Standardized diet compositions of *Carcharhinus* species. Prey abbreviations are explained in table S1.

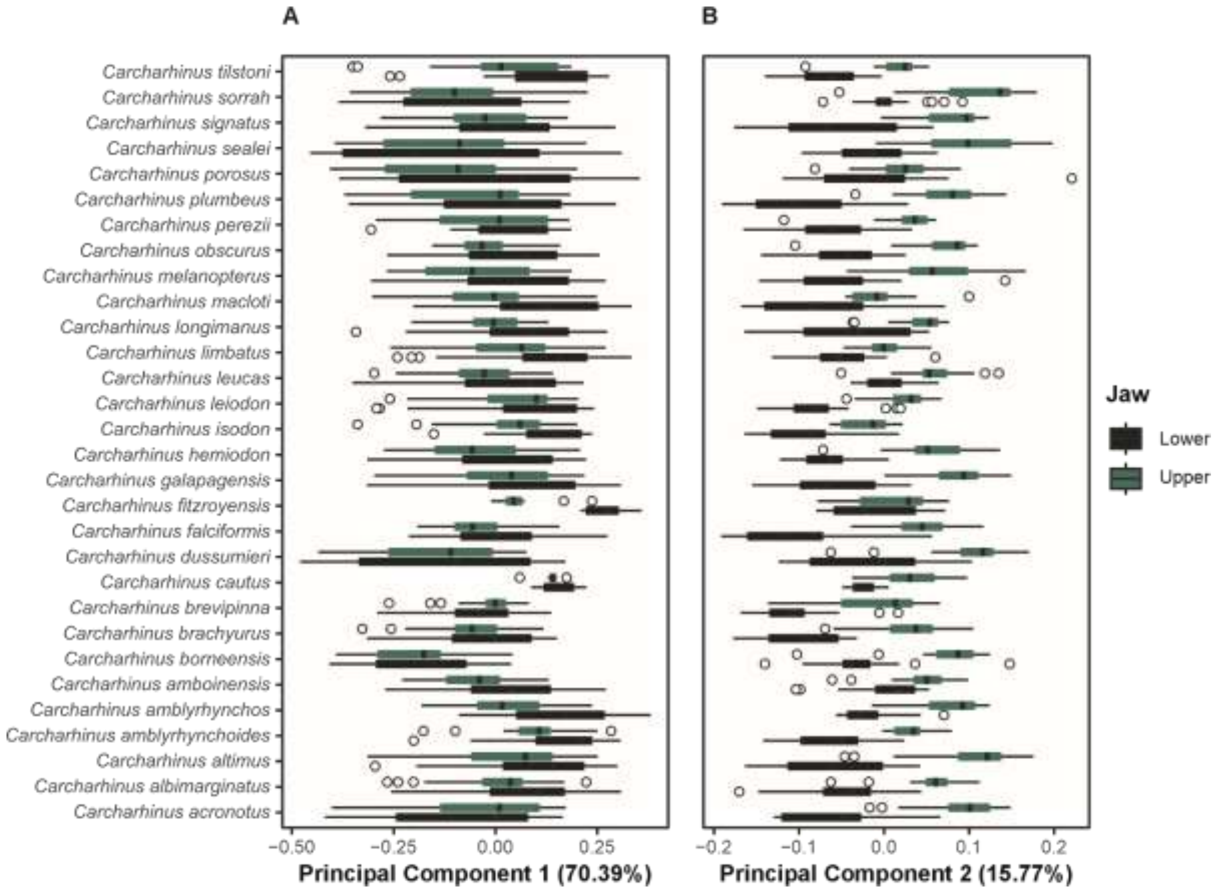

Fig. S11: Dignathic heterodonty in *Carcharhinus* species. Box-and-whisker plots depicting morphological variation along PC1 and PC2 for **(A)** upper and **(B)** lower jaw teeth. The median, 25th and 75th percentile hinges, and whiskers are visualized with specimen outliers (white points).

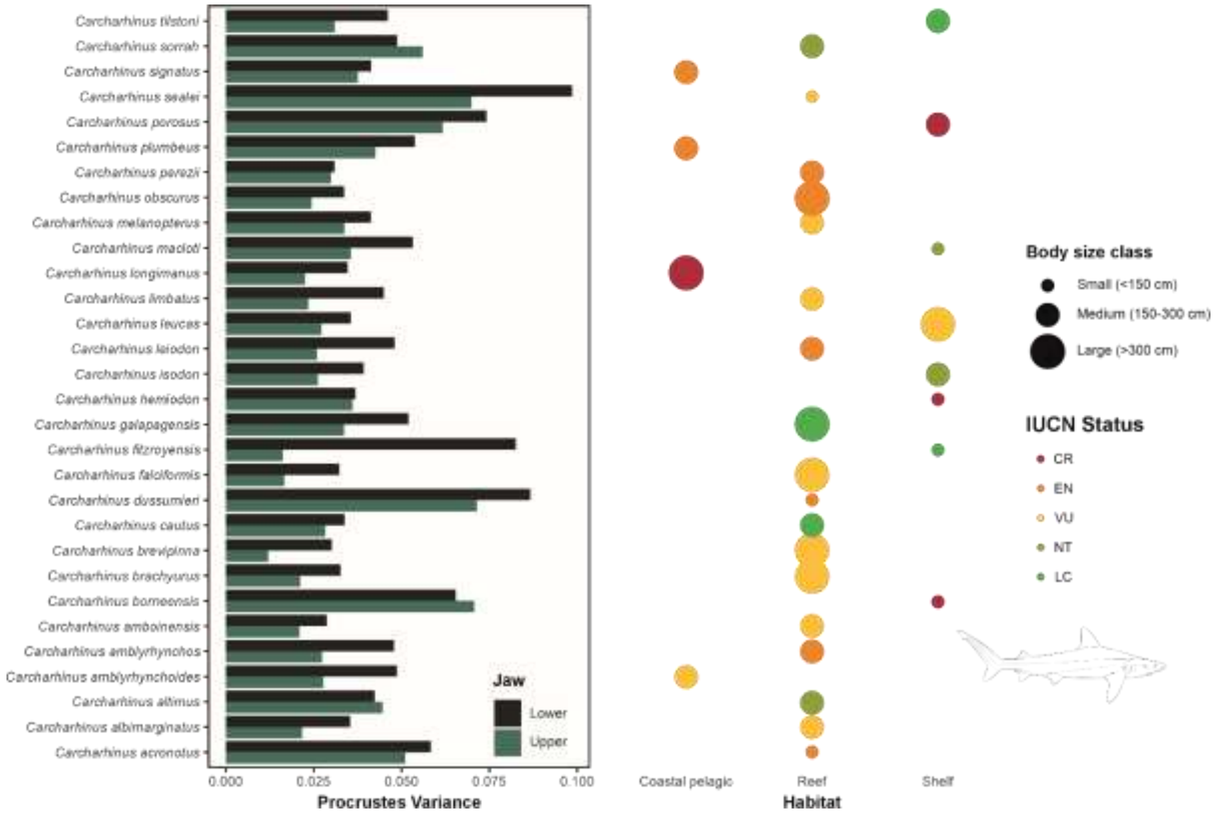

Fig. S12: Within-species dental disparity estimates. Bar-plot shows the disparity in lower and upper teeth within *Carcharhinus* species. In terms of absolute values, the variability of lower teeth is notably higher than upper teeth in *C. fitzroyensis* followed by *C. brevipinna*. Habitat, body size and the threat status of each species are provided for additional context. Line-drawing by Yameen Arshad and inspired by illustrations in Shark of the World (*49*).

Table S1. Description of prey categories for standardized diet compositions. The zooplankton prey-category was removed from the original dataset (75) as it did not apply to any *Carcharhinus* species.

Group code	Description
FISH	Teleost and agnathan fishes
CEPHALOPDS	Cephalopods (squids, octopuses)
MOLLUSUCS	Molluscs (excluding cephalopods)
DECAPODS	Decapod crustaceans (shrimps, crabs, prawns, lobsters)
OTHER.INVERTS	Other invertebrates (excluding molluscs, crustaceans, and zooplankton)
BIRDS	Seabirds
REPTILES	Marine reptiles (sea turtles and sea snakes)
MAMMALS	Marine mammals (cetaceans, pinnipeds, mustelids)
CHONDRICHTYANS	Chondrichthyan fishes (sharks, skates, rays, and chimaerids)
PLANTS	Plants (marine plants and algae)

Table S2. Procrustes ANCOVA results for the effects of log-transformed centroid-size, species identity, and dignathic heterodonty (with interaction) on tooth shape (n = 1256), based on 999 RRPP permutations. Coefficient estimation via ordinary least squares (OLS). Type I (sequential) sums of squares were used to calculate sums of squares and cross-products matrices. Effect sizes (Z) are based on F distribution.

	Df	SS	MS	R^2	F	Z	Pr(>F)
Log(Csize)	1	0.242	0.2423	0.00490	8.4582	2.9138	0.001
Species	29	8.649	0.2982	0.17493	10.4092	11.2770	0.001
Dignathic Heterodonty	1	4.415	4.4147	0.08929	154.0815	6.1504	0.001
Log(Csize):Species	29	1.898	0.0655	0.03840	2.2849	4.4224	0.001
Residuals	1195	34.239	0.0287	0.69249			
Total	1255	49.443					

Abbreviations: Df = degrees of freedom; SS = sequential sums of squares; MS = Mean squares; R^2 = coefficient of determination; F statistics = F value by permutation; Z = effect size. *P*-values are based on 999 permutations.

Table S3. Procrustes ANOVA results for the effect of IUCN conservation status on tooth shape (n = 1256) in *Carcharhinus* species, with significance assessed using 999 RRPP permutations. Coefficient estimation via ordinary least squares (OLS). Type I (sequential) sums of squares were used to calculate sums of squares and cross-products matrices. Effect sizes (Z) are based on F distribution.

	Df	SS	MS	R^2	F	Z	Pr(>F)
IUCN	4	1.077	0.269268	0.02178	6.9646	4.7913	0.001
Residuals	1251	48.366	0.038662	0.97822			
Total	1255	49.443					

Abbreviations: Df = degrees of freedom; SS = sequential sums of squares; MS = Mean squares; R^2 = coefficient of determination; F statistics = F value by permutation; Z = effect size. *P*-values are based on 999 permutations.
